# Wnt3a nanodisks promote ex vivo expansion of hematopoietic stem and progenitor cells

**DOI:** 10.1186/s12951-016-0218-5

**Published:** 2016-08-23

**Authors:** Nahal R. Lalefar, Andrzej Witkowski, Jens B. Simonsen, Robert O. Ryan

**Affiliations:** 1 Children’s Hospital Oakland Research Institute, 5700 Martin Luther King Jr. Way, Oakland, CA 94609 USA; 2 Department of Hematology/Oncology, UCSF Benioff Children’s Hospital Oakland, 747 52nd Street, Oakland, CA 94609 USA; 3 Department of Micro- and Nanotechnology, DTU Nanotech, Technical University of Denmark, Lyngby, Denmark

**Keywords:** Murine Wnt3a, Nanodisk, Reconstituted high-density lipoprotein, Hematopoietic stem and progenitor cell, β-Catenin, Lipid-modified proteins

## Abstract

**Background:**

Wnt proteins modulate development, stem cell fate and cancer through interactions with cell surface receptors. Wnts are cysteine-rich, glycosylated, lipid modified, two domain proteins that are prone to aggregation. The culprit responsible for this behavior is a covalently bound palmitoleoyl moiety in the N-terminal domain.

**Results:**

By combining murine Wnt3a with phospholipid and apolipoprotein A-I, ternary complexes termed nanodisks (ND) were generated. ND-associated Wnt3a is soluble in the absence of detergent micelles and gel filtration chromatography revealed that Wnt3a co-elutes with ND. In signaling assays, Wnt3a ND induced β-catenin stabilization in mouse fibroblasts as well as hematopoietic stem and progenitor cells (HSPC). Prolonged exposure of HSPC to Wnt3a ND stimulated proliferation and expansion of Lin^−^ Sca-1^+^ c-Kit^+^ cells. Surprisingly, ND lacking Wnt3a contributed to Lin^−^ Sca-1^+^ c-Kit^+^ cell expansion, an effect that was not mediated through β-catenin.

**Conclusions:**

The data indicate Wnt3a ND constitute a water-soluble transport vehicle capable of promoting ex vivo expansion of HSPC.

**Electronic supplementary material:**

The online version of this article (doi:10.1186/s12951-016-0218-5) contains supplementary material, which is available to authorized users.

## Background

Members of the Wnt protein family are morphogens that signal cell populations distant from their site of synthesis in a concentration-dependent manner. In canonical Wnt signaling, an anti-apoptotic cascade is initiated upon Wnt binding to cell surface co-receptors, the seven-pass transmembrane protein, frizzled, and closely related members of the low density lipoprotein receptor family, LRP 5/6 [1]. Upon engaging its co-receptors, Wnt triggers a series of intracellular events that lead to accumulation of β-catenin, which migrates to the nucleus and binds to the Tcf/Lef family of DNA binding-proteins, transiently converting them into transcriptional activators that induce target gene expression. Wnt signaling has been shown to be critical in hematopoietic stem cell homeostasis by inducing proliferation and reducing differentiation, thereby promoting functional self-renewal [2].

Each of the nineteen human Wnt proteins and a myriad of others from distant species share structural features that are essential to function. With few exceptions [3], these include a secretory signal sequence, multiple glycosylation sites, numerous cysteine residues that form disulfide bonds and a unique serine residue that serves as the site for covalent attachment of a long chain fatty acid [4]. Murine Wnt3a is fatty acylated with palmitoleic acid on serine 209 [5–7]. Replacement of this serine with another amino acid abolishes Wnt lipidation and interferes with intracellular processing and secretion of the protein [5–7].

Fatty acylation imparts hydrophobicity to Wnt proteins, reducing their aqueous solubility. This behavior may be explained by the highly exposed nature of this fatty acid within the context of the overall Wnt structure [8]. As a result, all acylated Wnt preparations require detergent micelles to maintain solubility and prevent aggregation. Whereas zwitterionic detergents, such as CHAPS (3-[(3-cholamidopropyl) dimethylammonio]-1-propanesulfonate), effectively confer solubility to isolated Wnt preparations, they can elicit negative effects on cell membrane integrity, potentially compromising studies of Wnt biological activity [9]. To circumvent this, strategies designed to maintain Wnt solubility in the absence of detergent micelles have been pursued. These include incorporating Wnt into liposomes [10], interaction with a lipocalin protein [11] or sequestration of the exposed fatty acid by β-methyl cyclodextrin [12].

**Fig. 1:**
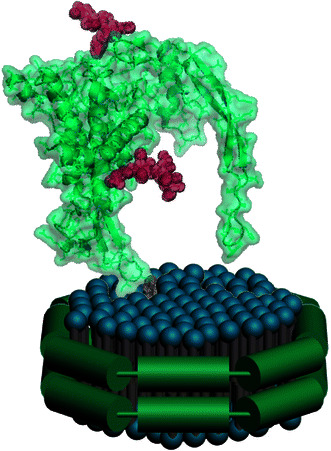
Model depiction of a Wnt ND. When combined, phospholipid and apoA-I interact to form discoidal rHDL complexes. When this reaction proceeds in the presence of Wnt3a, its covalently bound fatty acid is postulated to insert into the ND bilayer

In the present study, isolated recombinant murine Wnt3a has been incorporated into nanoscale reconstituted high-density lipoprotein (rHDL) particles, termed nanodisks (ND). ND are defined as rHDL formulated in the presence of an extraneous hydrophobic bioactive agent [13, 14] or transmembrane protein [15]. ND possess unique biological properties that distinguish them from classical rHDL [16]. Structurally, ND are disk-shaped ternary complexes of bilayer forming lipids, amphipathic protein scaffold and integrated hydrophobic bioactive agent or protein. The lipid moiety of ND is generally a phospholipid while the scaffold component may be a member of the class of exchangeable apolipoproteins [13], fragments thereof or a synthetic peptide [17]. As depicted in Fig. 1, it is hypothesized that Wnt3a association with the bilayer membrane of ND involves insertion of its fatty acyl chain into the lipid milieu. Thus, ND provide a membrane-like environment for Wnt3a transport akin to lipophorin particles that transport “Wingless”, the *Drosophila* homologue of Wnt [18]. Herein, we show that murine Wnt3a associates with ND in a manner that facilitates its presentation to target cell receptors in a biologically active state. Furthermore, the finding that Wnt3a ND induce self-renewal of a population of hematopoietic stem and progenitor cells (HSPC), suggest in vivo applications are feasible.

## Methods

### Proteins and lipids

Murine Wnt3a was expressed in stably transfected *Drosophila* S2 cells and isolated from conditioned media according to Witkowski et al. [12]. Human apolipoprotein (apo) A-I was expressed in *E. coli* and isolated as described elsewhere [19]. 1,2-dimyristoyl-*sn*-glycero-3-phosphocholine (DMPC) and 1,2-dimyristoyl-*sn*-glycero-3-phospho-(1′-rac-glycerol) (DMPG) were obtained from Avanti Polar Lipids Inc.

### Wnt3a ND formulation

Five milligram DMPC or a mixture of 3.5 mg DMPC and 1.5 mg DMPG were dissolved in chloroform–methanol (3:1, v/v) and dried under a stream of N_2_ gas, thereby coating the walls of a test tube with phospholipid. The sample was further dried under vacuum to remove residual organic solvent. The dried lipids were dispersed in 1 ml phosphate buffered saline (PBS; 20 mM sodium phosphate, pH 7.4, 150 mM NaCl) with vortexing and bath sonication. Whereas rHDL formation is induced by the addition of apoA-I to such phospholipid dispersions, for Wnt3a ND preparations isolated Wnt3a was introduced prior to the addition of apoA-I. Two hundred to nine hundred nanogram of Wnt3a (in 1 % CHAPS) was added to 200 µg DMPC or DMPC/G mixture (final volume = 227 µl). The final concentration of CHAPS was not higher than 1.04 mM, well below its critical micelle concentration (~6 mM). The sample was incubated at 27 °C for 1 h with shaking followed by the addition of 80 µg apoA-I (final volume = 250 µl) with continued incubation at 24 °C for 45 min. Control incubations lacking phospholipid, apoA-I or Wnt3a were performed in parallel, with all final sample volumes remaining identical. The samples were then centrifuged at 10,000×*g* for 6 min at 4 °C and the recovered supernatant stored at 4 °C. In some cases, Wnt3a solubility was assessed by centrifugation at 25,000×*g* for 30–45 min at 4 °C (TL100.2 rotor, Optima TL Ultracentrifuge, Beckman). Following centrifugation, a portion of the supernatant was subjected to anti-Wnt3a immunoblot analysis.

### Size exclusion chromatography

Wnt3a ND (250 µl) were concentrated by centrifugal filtration (Centricon 50 kDa MWCO) to 70 µl and subjected to HPLC on a 9.4 × 250 mm Zorbax GF-250 column equilibrated in PBS plus 0.15 M NaCl. Chromatography was performed on a Perkin-Elmer Series 200 System at a flow rate of 1 ml/min. A portion (150 µl) of selected fractions was precipitated with chloroform/methanol after addition of 10 µg bovine serum albumin [20]. Pelleted material was solubilized in electrophoresis buffer and subjected to anti-Wnt3a immunoblot.

### Immunoblot analysis

Proteins were separated by sodium dodecyl sulfate polyacrylamide gel electrophoresis, transferred to polyvinylidene fluoride membrane and probed with rabbit anti-mouse Wnt3a (1:6000 dilution, Abcam). Positive bands were detected with a horseradish peroxidase (HRP)-conjugated anti-rabbit IgG antibody (1:5000 dilution, Jackson Immuno Research). The amount of Wnt3a present in samples was calculated from a standard curve generated with isolated Wnt3a [12].

### Wnt3a signaling activity

Assays of canonical Wnt3a signaling were conducted according to Hannoush [21]. Briefly, mouse fibroblasts (LS/L cells) were plated in Dulbecco’s Modified Eagle’s medium (DMEM)/10 % fetal bovine serum (FBS) in a 96-well clear bottom, black walled plate at 25,000–50,000 cells/well and incubated overnight at 37 °C, 5 % CO_2_. Specified Wnt3a samples were added to the wells (80–100 % confluence) in a dilution series and incubated for 16–18 h. The cells were fixed in 4 % paraformaldehyde for 1 h and washed three times with PBS (50 µl/well). Cells were permeabilized with PBS/0.1 % Triton X-100 (50 µl/well) and Odyssey^®^ blocking buffer (LI-COR, #927-40000) was added (50 µl/well). After 2 h, the buffer was replaced with anti-mouse β-catenin (1:200; BD, #610154) in LI-COR blocking buffer (20 µl/well), incubated overnight at 4 °C and washed three times with PBS/0.1 % Tween-20. Infrared anti-mouse IRDye800CW secondary antibody (1: 200, Rockland Antibodies and Assays #610-131-003) and DRAQ5 Fluorescent probe (1: 10,000, Thermo Scientific, #62254) in PBS/0.5 % Tween-20 were then added (20 µl/well). The plates were incubated for 1 h at 22 °C, the wells washed three times with PBS/0.1 % Tween-20 and supplemented with PBS (50 µl/well). The plates were covered with black seals and imaged on an Odyssey infrared scanner using both the 700 and 800 nm wavelength channels. Data were acquired using Odyssey software, exported and analyzed using Excel. β-Catenin values were background subtracted from wells treated with secondary antibody only and normalized to total DNA fluorescence signal. Activities were calculated from the slope of the linear portion of normalized β-catenin values versus the logarithm of Wnt3a concentration. Assays were performed in triplicate.

### Isolation of HSPC

Murine stem and progenitor cell harvest protocol approval was obtained from the Institutional Animal Care and Use Committee at Children’s Hospital Oakland Research Institute. C57BL/6J mice (Jackson Laboratories) between 6 and 12 weeks of age were sacrificed by CO_2_ asphyxiation. Whole bone marrow was harvested by crushing the femurs/tibia/humeri/pelvic bones in sterile PBS without calcium or magnesium, supplemented with 2 % FBS. Red blood cells were lysed in 0.15 M NH_4_Cl, 10.0 mM KHCO_3_, 0.1 mM EDTA and incubated on ice for 3–5 min. Bone marrow derived cells were separated by ficoll-paque density gradient (1.077 g/ml, LymphoPrep, Stemcell Technologies) to isolate mononuclear cells. Mononuclear cells were then incubated with anti-CD117 (c-kit)-labeled magnetic microbeads on ice for 30 min. A magnetic LS column with MACS separator (Miltenyi Biotec) was used to collect c-kit^+^ mononuclear cells according to the manufacturer’s protocol. Lineage staining was performed with a cocktail of biotinylated anti-mouse antibodies to Mac-1 (CD11b), Gr-1 (Ly-6G/C), Ter119 (Ly-76), CD3e, and B220 (CD45R) (BioLegend). For detection or sorting, c-Kit-APC, Sca-1-PE-Cy7 and streptavidin conjugated to APC-Cy7 (BioLegend) were employed. Zombie UV Fixable viability kit was used for dead cell exclusion. A population of Lin^−^ Sca-1^+^ c-kit^+^ (LSK) cells was obtained by analysis and sorting on a FACSAria (Becton–Dickinson). Data analysis was performed using FlowJo v10 and BD FACSDIVA software.

### Wnt3a mediated effects on cellular β-catenin levels

LSK cells were seeded and cultured (1000 cells per well) as described below in the presence or absence of an indicated Wnt3a formulation. After 24 h cells from 12 wells were combined, washed with PBS, lysed in RIPA buffer containing protease inhibitors and subjected to immunoblot analysis to quantify β-catenin (mouse β-catenin monoclonal antibody, 1:2000, BD Transduction Laboratories). Glyceraldehyde-3-phosphate dehydrogenase (GAPDH) was used for normalization (mouse GAPDH monoclonal antibody, 1:4000, Ambion). In both cases, signal was detected with ECL (Advansta) reagent after incubation with HRP-conjugated anti-mouse IgG (1:10,000, Pierce).

### LSK cell expansion assays

One thousand sorted LSK cells were cultured in low adherence, 96-well round bottom plates with 200 µl StemSpan media, 10 % FBS, 100 U/ml penicillin, 100 µg/ml streptomycin and 25 ng/ml stem cell factor (SCF, Peprotech). Where indicated, the medium was supplemented with 100 ng Wnt3a (as Wnt3a CHAPS micelles, Wnt3a ND or Wnt3a phospholipid). In control incubations, cells were incubated with PBS (alone or supplemented with a corresponding amount of 1 % CHAPS) or empty ND. Every 48 h one half of the medium was exchanged with fresh medium (±Wnt3a formulation). After 6 days, cells were collected, counted by hemocytometry and analyzed by FACS on a BD LSRFortessa analyzer (Additional file 1: Figure S1 for a representative example). Lineage staining was performed with a cocktail of biotinylated anti-mouse antibodies as described above. For detection, c-Kit-APC, Sca-1-PE-Cy7 and streptavidin conjugated to FITC were employed. Zombie violet Fixable viability kit was used for dead cell exclusion.

### Statistics

Values reported are the average of an indicated number of experiments and errors are standard errors of the mean. Paired, two-tailed Student’s t test was used to calculate statistical significance.

## Results

### Effect of Wnt3a and apoA-I on phospholipid light scattering intensity

**Fig. 2:**
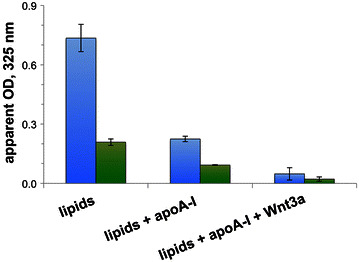
Effect of apoA-I and Wnt3a on phospholipid dispersion light scattering intensity. DMPC (*blue columns*) and DMPC/DMPG (70:30, w/w, *green columns*) were dispersed in PBS (0.6 mM final lipid concentration). The following were added to these lipid dispersions: PBS (*left*), apoA-I (*middle*) or apoA-I + Wnt3a (*right*). In the latter two cases the apoA-I/lipid mass ratio was 1:2.5. Following incubation, sample light scattering intensity was measured at 325 nm on a Perkin Elmer LS20 UV/Vis spectrophotometer. Values reported are the average of two to three independent preparations

When dispersed in PBS, the glycerophospholipid DMPC manifests an opaque, turbid appearance with significant light scattering intensity (Fig. 2). As shown previously [13, 22], addition of apoA-I to a DMPC dispersion induces formation of nanoscale-sized rHDL with reduced light scattering intensity. Inclusion of Wnt3a did not interfere with apoA-I-mediated transformation of DMPC into rHDL. The finding that Wnt3a containing reactions gave rise to final light scattering intensity values lower than those containing apoA-I and phospholipid may be due to the presence of CHAPS monomers in these samples. In all cases, when a mixture of DMPC and DMPG (70:30 w/w) was employed, although the initial light scattering intensity was lower, similar results were obtained.

### Wnt3a ND characterization

**Fig. 3:**
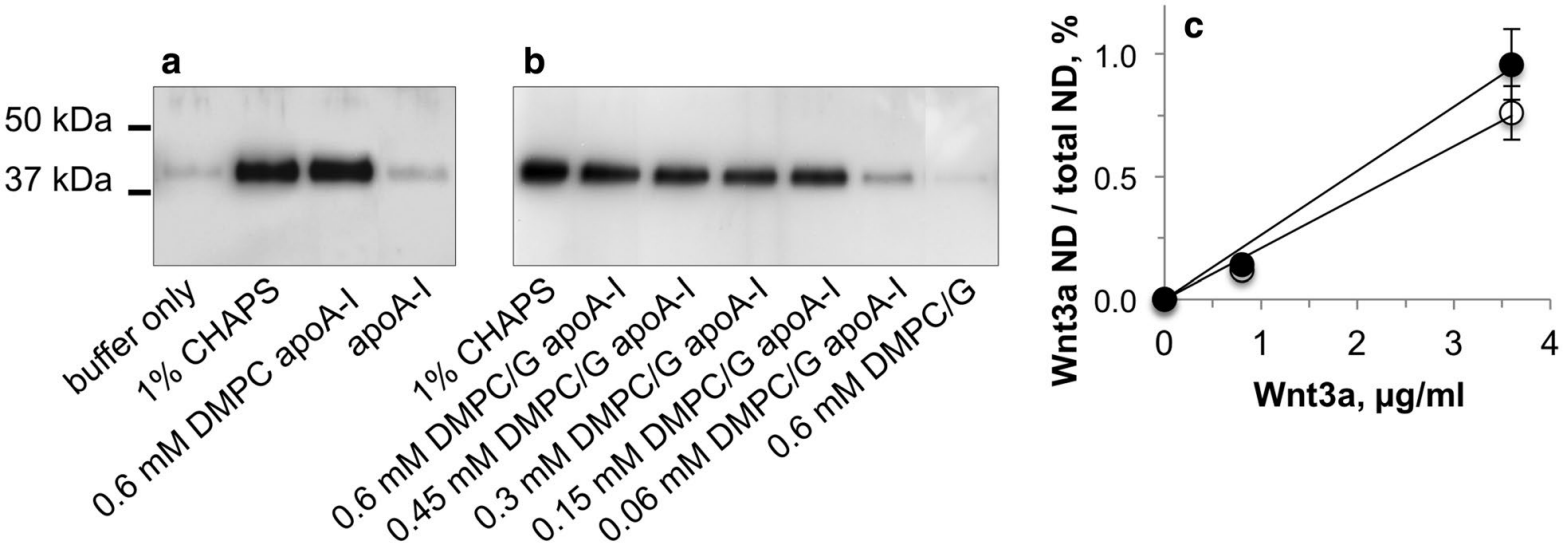
Aqueous solubility of Wnt3a formulations. A stock solution of Wnt3a in 1 % CHAPS was diluted into buffer solutions containing the indicated concentrations of lipids and centrifuged at 25,000×*g* for 30 min (**a**) or 25,000×*g* for 45 min (**b**). A portion of each supernatant was analyzed by anti-Wnt3a immunoblot. The mass ratio of lipid to apoA-I was maintained at 2.5:1 and in all cases the Wnt3a concentration was 0.8 µg/ml. **c** Plot of Wnt3a concentration dependent binding to ND. The amount of Wnt3a bound to ND was determined by quantitative immunoblot and ND concentration was calculated assuming two apoA-I per ND. *Open circles* DMPC; *closed circles* DMPC/DMPG. Values reported are the average of two to five independent experiments

Previous studies have shown that, when ND formation is induced in the presence of hydrophobic bioactive agents or transmembrane proteins, the added components become embedded in the lipid milieu of the product particles [14]. The term ND was coined to distinguish these ternary complexes from binary rHDL. To investigate whether Wnt3a physically associates with rHDL to form Wnt3a ND, an ultracentrifugation assay was performed. Upon dilution of Wnt3a CHAPS stock solution below the critical micelle concentration of CHAPS, very little Wnt3a is recovered in the supernatant following centrifugation (Fig. 3a). By contrast, when Wnt3a CHAPS stock solution was diluted into PBS containing CHAPS (1 % w/v), Wnt3a was recovered in the supernatant. Similarly, when apoA-I was added to Wnt3a and phospholipid to induce ND formation, Wnt3a was recovered in the supernatant. Based on quantitative immunoblot analysis, 46 ± 7 and 61 ± 8 % of the starting Wnt3a was recovered in the supernatant of DMPC- and DMPC/DMPG- derived ND, respectively. By contrast, when Wnt3a was added to an aqueous dispersion of DMPC without apoA-I, Wnt3a pelleted upon ultracentrifugation [10].

Attempts to increase the amount of Wnt3a incorporated into a given ND preparation were impeded by the limited solubility of Wnt3a and the amount of CHAPS present in the Wnt3a stock solution. At a constant Wnt3a concentration, as the amount of phospholipid and apoA-I in the reaction mix is was reduced, less Wnt3a was recovered in the supernatant (Fig. 3b). When the amount of Wnt3a was varied at a fixed phospholipid/apoA-I concentration (Fig. 3c), however, a linear relationship was observed between the amount of Wnt3a added and the proportion recovered in ND. Thus, the final working solution of Wnt3a ND used in experiments also contains “empty” ND. Under these conditions, however, Wnt3a is protected against aggregation and remains soluble in aqueous solution.

**Fig. 4:**
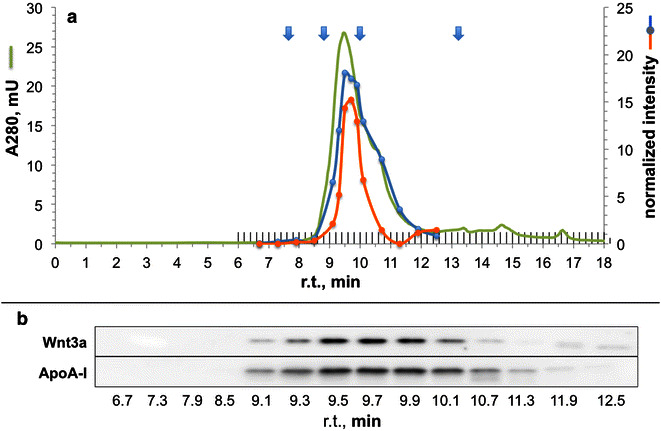
Gel filtration chromatography of Wnt3a ND. A Wnt3a ND preparation was subjected to size exclusion chromatography with collection of 1 ml fractions (**a**). The elution position of standards (670, 132, 66 and 0.4 kDa) is depicted by *arrows* and the absorbance 280 profile for Wnt3a ND elution is shown in *green*. The data are representative of two independent experiments. A portion of selected fractions was analyzed by immunoblot (**b**) by probing with anti-Wnt3a (see *orange line* in **a**) and anti-apoA-I (see *blue line* in **a**)

The size and stability of Wnt3a ND were then examined by gel filtration chromatography (Fig. 4). Immunoblot analysis of a portion of selected column fractions revealed that Wnt3a and apoA-I co-elute from the column with an apparent molecular weight in the range of 100 kDa. This value is consistent with previous studies of ND [17, 23–25] wherein evidence of discoidal morphology was obtained by electron microscopy or atomic force microscopy. By contrast, when a control sample containing apoA-I and Wnt3a, but no phospholipid, was chromatographed, apoA-I was recovered in the column eluate but Wnt3a was lost (data not shown).

### Canonical Wnt signaling activity in murine fibroblasts

**Fig. 5:**
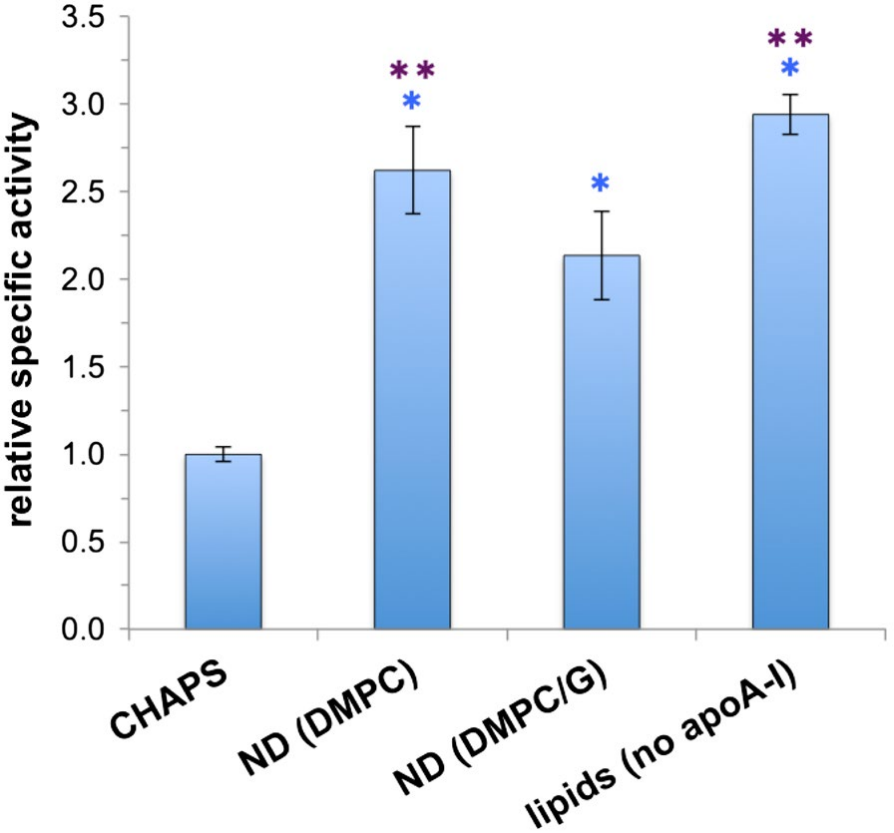
Relative ability of Wnt3a formulations to activate canonical Wnt signaling in mouse fibroblasts. Specified Wnt3a formulations were incubated with mouse fibroblasts for 16–18 h and, following incubation, the cells were subjected to immunofluorescence assay of β-catenin and reported as relative specific activity. *Single* and *double asterisks* indicate p < 0.001 and p < 0.02 by the Student’s t test when compared to Wnt3a CHAPS and Wnt3a ND (DMPC/DMPG) preparations, respectively. n = 5 for each Wnt3a ND preparation and n = 3 for average of both Wnt3a DMPC and Wnt3a DMPC/DMPG (no apoA-I)

To compare the biological activity of different Wnt3a formulations, murine fibroblasts were incubated with Wnt3a CHAPS micelles, Wnt3a ND or Wnt3a phospholipid vesicles. Canonical Wnt signaling activity was measured by quantitative infrared immunofluorescence assay of cellular β-catenin levels. Wnt3a ND (DMPC) was 2.6 ± 0.3 times more active than Wnt3a CHAPS (Fig. 5) while Wnt3a ND (DMPC/DMPG) was 2.1 ± 0.3 times more active. Comparable values were obtained with Wnt3a phospholipid vesicles (no apoA-I). ND prepared without Wnt3a had no effect on fibroblast β-catenin levels.

### Effect of Wnt3a ND on β-catenin levels in LSK cells

**Fig. 6:**
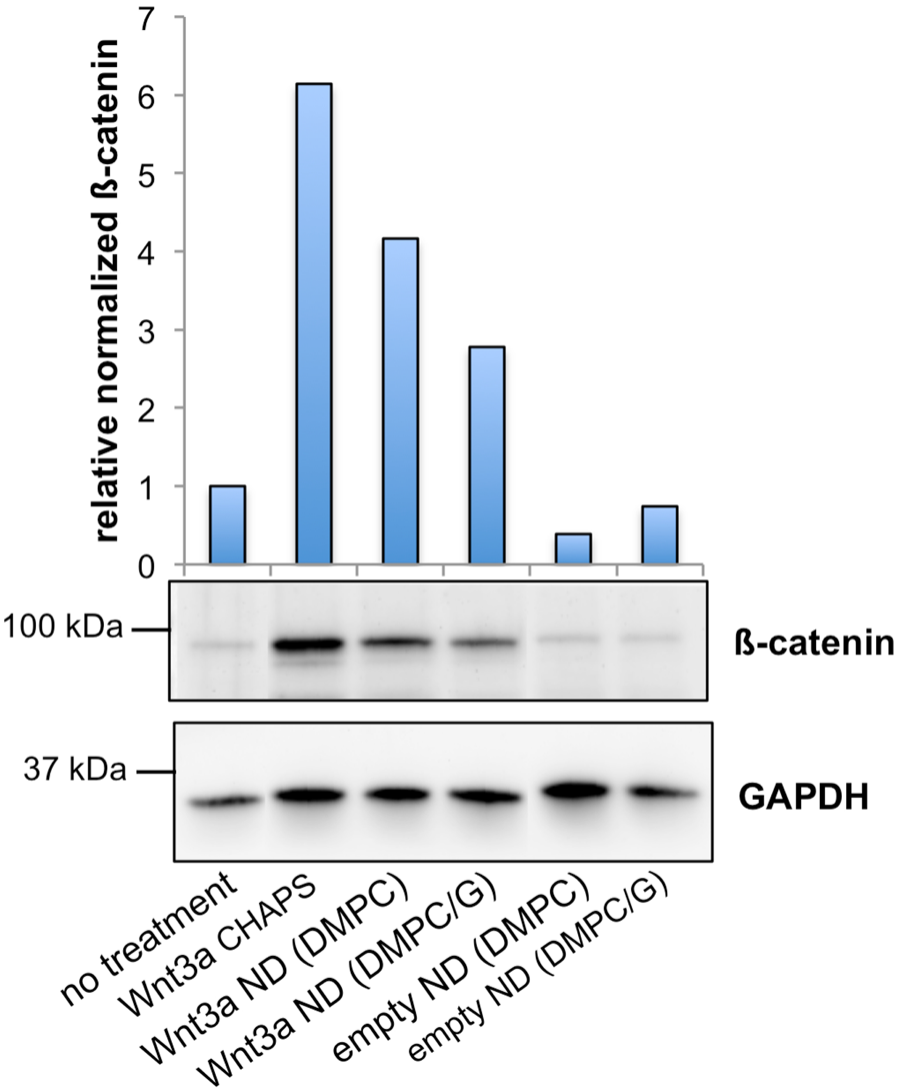
Effect of Wnt3a formulations on β-catenin levels in LSK cells. LSK cells were incubated with specified Wnt3a preparations as described in the “Methods” section at a final Wnt3a concentration of 100 ng/ml. After 24 h the cells were collected, lysed and subjected to anti β-catenin or anti GAPDH (loading control) immunoblot, A digitized and normalized histogram depicting the relative intensity of the β-catenin signal is presented (*upper panel*). The data reported are representative of four independent experiments

To determine the effect of Wnt3a ND on signal transduction in murine HSPC, LSK cells were isolated and incubated with Wnt3a CHAPS or Wnt3a ND for 24 h. Following incubation the cells were lysed and β-catenin content measured by immunoblot. In control incubations of PBS plus CHAPS (no Wnt3a), β-catenin levels were very low (Fig. 6). Likewise, incubation with empty ND had no measurable effect on β-catenin levels. By contrast, when LSK cells were incubated with Wnt3a CHAPS or Wnt3a ND, the cellular content of β-catenin increased, thereby indicating these Wnt3a formulations activate canonical Wnt signaling.

### Effect of Wnt3a ND on LSK cell proliferation/expansion ex vivo

**Fig. 7:**
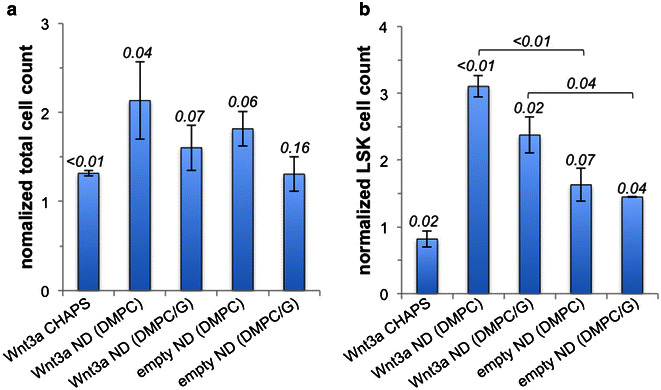
Effect of Wnt3a formulations on LSK cell proliferation/self-renewal. One thousand LSK cells were seeded per well and cultured as described. After 6 days, three wells were combined, counted and analyzed by flow cytometry. Values reported are normalized to cells cultured in medium supplemented with buffer only (for empty ND) or buffer containing equivalent amount of CHAPS (for Wnt3a ND and Wnt3a CHAPS). **a** Total cell count by hemocytometry. Control wells contained 87,000 ± 6000 (buffer only) and 76,000 ± 6000 (buffer with CHAPS) cells per well. **b** The LSK cell count obtained by FACS analysis [final numbers for control were 4690 ± 780 (buffer only) and 3650 ± 520 (buffer with CHAPS)]. p values (versus control) are shown above each *column*. The data present averages of four independent experiments

Given the effect of Wnt3a on LSK cell β-catenin levels, the effect of longer-term incubations with Wnt3a ND on proliferation/expansion of the LSK cell pool size was examined. All incubations included SCF, which was required for cell survival over the six-day culture period. At the end of the experiment, total cell proliferation was assessed by hemocytometry (Fig. 7a). Compared to LSK cells incubated with buffer, Wnt3a CHAPS and Wnt3a ND (DMPC) increased total cell proliferation. The cells were then analyzed by FACS to assess the extent of LSK cell expansion versus proliferation with differentiation. Compared to cells treated with buffer, Wnt3a CHAPS did not change the LSK cell pool size (Fig. 7b). On the other hand, both Wnt3a ND preparations (DMPC and DMPC/DMPG) increased the LSK cell pool size compared with Wnt3a CHAPS and buffer-CHAPS incubations. Unexpectedly, compared to incubations with the corresponding buffer, incubations with empty NDs (i.e. no Wnt3a) increased cell proliferation and LSK cell pool size. Hence, the response of isolated LSK cells to Wnt3a ND appears to be a cumulative effect of Wnt3a and the ND vehicle itself (i.e. rHDL). Although the underlying mechanism whereby empty ND can induce LSK cell expansion is unclear, evidence (Fig. 6) indicates it is not mediated through β-catenin.

## Discussion

The use of rHDL to solubilize extraneous hydrophobic biomolecules has expanded rapidly in recent years. The fact that these discoidal particles possess a planar phospholipid bilayer has led to their use as miniature membranes for integration of transmembrane proteins in a native like environment [26]. Likewise, rHDL have been used to solubilize a host of small hydrophobic bioactive compounds including amphotericin B, all *trans* retinoic acid, curcumin [14], simvastatin [27], a synthetic cationic lipid [28] and cardiolipin [29]. To distinguish these complexes from classical rHDL, the term ND is used. A potential ND interaction partner that has not been explored is the large family of proteins that are modified by covalent lipid attachment. This ubiquitous protein modification encompasses nearly 1000 proteins with wide diversity in both structure and function. Among the lipid moieties known to attach to proteins are fatty acids, isoprenoids, sterols and phospholipids. One such lipid modified protein, whose X-ray structure has been solved [8], is the Wnt morphogen. Wnt proteins possess a fatty acyl chain that protrudes prominently from one end of the protein and is considered largely responsible for the detergent micelle requirement to maintain solubility of isolated Wnt in buffer. Given the general property that lipid modified proteins are attracted to membranes, we hypothesized that Wnt may form a stable interaction with rHDL, thereby creating a Wnt ND. The data presented show that, when rHDL are formulated in the presence of isolated recombinant murine Wnt3a, it is conferred with aqueous solubility in the absence of detergent micelles and co-elutes with apoA-I following gel filtration chromatography. Whereas the classical “detergent dialysis” method [15, 22] commonly used to generate transmembrane protein-integrated ND gave a poor yield of Wnt3a ND (data not shown), the direct solubilization method [13] was successfully employed.

In a fibroblast-based assay, Wnt3a ND displayed enhanced biological activity compared to Wnt3a detergent micelles. Likewise, when incubated with freshly isolated HSPC (LSK cells), Wnt3a ND induced β-catenin stabilization, a measure of canonical Wnt signaling. These data indicate that association of Wnt3a with ND does not interfere with its ability to form a productive interaction with its co-receptors, frizzled and LRP 5/6, on the plasma membrane of target cells. Whereas it is most likely that Wnt3a association with ND involves fatty acyl chain insertion into the ND bilayer, it is also evident that this interaction is reversible since Wnt3a ND manifest biological activity. The results of centrifugation experiments and gel filtration chromatography support the conclusion that Wnt3a ND exist as a soluble, relatively stable complex. Thus, in cell-based Wnt signaling assays, it may be considered that dissociation of Wnt3a from ND is induced by exposure to the cell membrane or protein constituents therein and, upon exposure, the fatty acyl moiety is able to engage the frizzled receptor’s cysteine rich domain (CRD). Given the differential effects of Wnt3a CHAPS versus Wnt3a ND on LSK cells expansion in long-term cultures (6 days), it is conceivable that Wnt3a ND provide a more stable environment for Wnt3a such that sustained pathway activation is achieved. If so, this may provide an explanation for the observation that, in short-term incubations with LSK cells (Fig. 6), Wnt3a CHAPS activated β-catenin to a greater extent than Wnt3a ND.

A major advance toward understanding the structural properties of Wnt proteins occurred with the report by Janda et al. [8] that the palmitoleoyl moiety of *Xenopus* Wnt8 is highly exposed at one end of the protein and penetrates into a hydrophobic pocket of frizzled CRD. Insofar as the frizzled CRD employed in this study lacks the transmembrane segments of full length frizzled, this domain provides a soluble Wnt8 binding module that effectively prevents Wnt aggregation, allowing for crystallization and X-Ray structure determination of the complex. A curious finding from this study is that the N-terminal domain of Wnt8, to which the fatty acid is bound, contains a “saposin-like” fold. Saposins are a well-known family of proteins that adopt a compact helix bundle molecular architecture. At the same time, saposins possess lipid surface seeking behavior and in certain cases, membrane lytic activity [30].

Based on the structure of *Xenopus* Wnt8, it does not appear that monomeric Wnt is capable of self-sequestering its palmitoleoyl moiety from solvent exposure. This implies that, following secretion from cells, Wnts are constantly in contact with membranes and/or specific transport proteins. This contrasts with other lipid modified proteins, such as recoverin. Studies of this calcium-myristoyl switch protein [31] revealed its N-terminal myristoyl moiety is sequestered in an internal cleft and is exposed by a calcium-triggered conformational change. Given current knowledge, a similar model cannot be invoked for Wnt. As a result, isolated Wnt3a is prone to self-association or precipitation in the absence of detergent micelles [9, 12]. Given that Wnt’s ability to function as a morphogen requires transit through aqueous media, the general consensus is that it interacts with transport partners while navigating to its ultimate destination. A common feature of all such partners is an ability to protect the hydrophobic fatty acyl moiety from exposure to the aqueous surroundings. Lipoproteins, lipocalins, liposomes, cyclodextrins and frizzled CRD each provide a unique surface/cleft that can accommodate a fatty acid. Likewise, detergent micelles undoubtedly serve a similar purpose.

If it is assumed that the fatty acyl moiety is exposed, yet cannot be protected by the Wnt molecule to which it is attached, it is plausible to invoke lipoprotein binding or alternate protein-mediated interactions to maintain solubility. A conundrum emerges however, because the sequestered fatty acyl moiety is crucial for Wnt interaction with frizzled CRD and subsequent signal transduction. This implies that Wnt exists in equilibrium with transport partners yet frizzled CRD binding is the favored interaction partner. How the fatty acyl moiety of Wnt successfully gains access to frizzled CRD, however, remains an unanswered question. Insofar as both frizzled and LRP 5/6 are transmembrane proteins, membrane contact by Wnt could play a role. For example, contact of the saposin-like subdomain with target cell membranes could induce conformational “opening” of its helix bundle, thereby exposing a cryptic membrane interaction site. Given the close proximity of the palmitoleic acid moiety and the saposin-like fold, conformational change in this segment of the protein may alter the orientation of this fatty acid. If membrane binding via the saposin-like fold promotes Wnt dissociation from a Wnt transport/binding partner, then the fatty acyl chain could become available to interact with the membrane or perhaps, frizzled CRD. In this case, following engagement of frizzled, it is conceivable that the C-terminal cytokine-like domain of Wnt becomes positioned where interaction with LRP 5/6 is favored. Once these co-receptors are engaged, canonical Wnt signal transduction proceeds.

In the present study we have demonstrated that murine Wnt3a interacts with ND. Advantages of ND include nanoscale size, solubility in aqueous media and an apparent ability to release bound Wnt upon presentation to cell surface receptors. It is likely that Wnt association with ND is dependent upon fatty acyl chain interaction with the phospholipid bilayer surface. By penetrating into the ND bilayer, the Wnt fatty acyl chain is effectively protected from aqueous exposure, thereby maintaining Wnt in a soluble, monomeric state.

Compared to Wnt3a CHAPS micelles, Wnt3a ND (formulated with DMPC or DMPC/DMPG) induced an increase in the number of LSK cells by twofold to threefold. The effect of Wnt3a ND (DMPC) on LSK cell numbers compared to Wnt3a ND (DMPC/DMPG) was similar to their relative abilities to induce canonical Wnt signaling in mouse fibroblasts. Consistent with this, the data showed that incubations of Wnt3a ND with HSPC resulted in stabilization of β-catenin (i.e. canonical Wnt signaling). Importantly, in vitro and in vivo studies indicate high Wnt signaling activity can drive differentiation of stem cell populations [32–34]. These findings suggest that maximal stem cell expansion requires an optimal level of β-catenin and that over stimulation by excess Wnt or other factors can increase differentiation.

Further confounding the goal of expanding stem cells is the observation that, although empty ND had no effect on β-catenin levels in HSPC, they did elicit a positive effect on cell proliferation and LSK cell pool size. Thus, the effect of Wnt3a ND on LSK cell expansion is a cumulative effect of Wnt3a and the ND (rHDL) vehicle itself. Given the unexpected nature of this finding, we examined the literature for precedent. In one case, Feng et al. [35] reported that low-density lipoprotein (LDL) promotes HSPC proliferation and increased differentiation to monocytes and neutrophils. At the same time, these authors observed that inclusion of HDL inhibited LDL-induced HSPC proliferation in vitro. Although these data infer that HDL exerts an inhibitory effect on HSPC proliferation, the authors did not examine the effect of HDL directly. However, on the basis of in vivo experiments wherein rHDL was administered to *ldlr* (−/−) mice, a reduction in LSK cells was reported.

Others have speculated that HDL binding to scavenger receptor type B1, via interaction with apoA-I, may regulate HSPC [36]. In keeping with this, in vivo administration of apoA-I led to fewer LSK cells in wild type mice than *scarb1* (−/−) mice, when fed a high fat diet. In another study employing genetically engineered mice, a combined deficiency of *abca1* and *abcg1* was associated with increased LSK cell numbers, an effect that was suppressed when these mice were crossed with apoA-I transgenic mice [37]. On the basis of these results, it may be implied that HDL (or rHDL) exert a negative effect on HSPC self-renewal. While our results show an increase in LSK cell numbers when HSPC are incubated with rHDL in vitro, experimental differences make direct comparisons difficult. For example, no previous studies have examined the effect of rHDL on HSPC self-renewal in vitro. Many factors, including the use of genetically modified mouse strains and the complexity of the in vivo setting may affect the outcome. As such, additional experiments will be required to fully characterize the effect of reconstituted or native HDL on HSPC self-renewal in vivo and in vitro. At the same time, the data presented herein support the conclusion that Wnt3a ND are capable of inducing HSPC expansion ex vivo for use in stem cell therapy applications.

## Conclusions

Recombinant murine Wnt3a has been stably incorporated into apolipoprotein-stabilized nanoscale disk shaped lipid particles termed nanodisks (ND). Wnt3a and the apolipoprotein scaffold protein co-elute when Wnt3a ND are subjected to gel filtration chromatography. Wnt3a ND activate canonical Wnt signaling in cultured fibroblasts and induce expansion of hematopoietic stem and progenitor cells ex vivo. This study is the first report of lipid modified protein incorporation into ND.

### Additional file

**Additional file 1: Figure S1.** Flow cytometry analysis of LSK cell proliferation. One thousand LSK cells were seeded into the wells of a culture plate. Following 6 days incubation under various conditions, cells from three wells of each condition were combined and analyzed by FACS. Individual plots, representative of an experiment conducted on four separate occasions, are shown. Negative lineage cells (positive for both Sca-1-PE-Cy7 and c-Kit-APC) are indicated by the box.

## Abbreviations

rHDL: reconstituted high-density lipoprotein
CHAPS: 3-[(3-cholamidopropyl) dimethylammonio]-1-propanesulfonate
ND: nanodisk
HSPC: hematopoietic stem and progenitor cell
Apo: apolipoprotein
DMPC: 1,2-dimyristoyl-*sn*-glycero-3-phosphocholine
DMPG: 1,2-dimyristoyl-*sn*-glycero-3-phospho-(1′-rac-glycerol)
DMPC/G: DMPC/DMPG
PBS: phosphate buffered saline
DMEM: Dulbecco’s Modified Eagle’s medium
FBS: fetal bovine serum
LSK: Lin^−^ Sca-1^+^ c-kit^+^
GAPDH: glyceraldehyde-3-phosphate dehydrogenase
SCF: stem cell factor
CRD: cysteine rich domain
LDL: low-density lipoprotein
