# Supplementary figures and images for: Wnt3a nanodisks promote ex vivo expansion of hematopoietic stem and progenitor cells
Source: J Nanobiotechnology. 2016 Aug 23;14:66. doi: 10.1186/s12951-016-0218-5 (PMC4995738; doi:10.1186/s12951-016-0218-5)

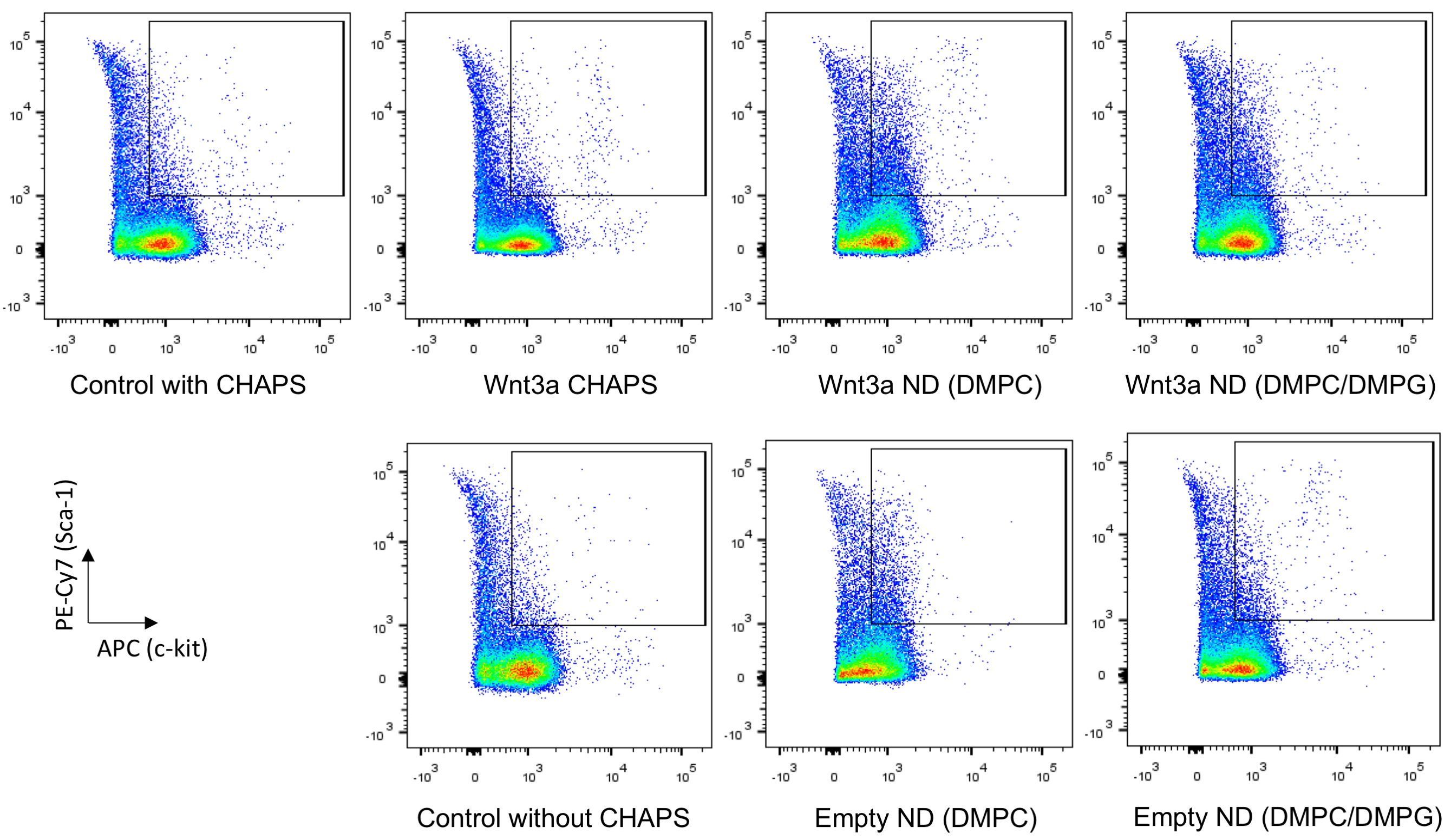

Supplement: Supplementary file 1 — Additional file 1: Figure S1. Flow cytometry analysis of LSK cell proliferation. One thousand LSK cells were seeded into the wells of a culture plate. Following 6 days incubation under various conditions, cells from three wells of each condition were combined and analyzed by FACS. Individual plots, representative of an experiment conducted on four separate occasions, are shown. Negative lineage cells (positive for both Sca-1-PE-Cy7 and c-Kit-APC) are indicated by the box. [file 12951_2016_218_MOESM1_ESM.jpg]
